# STICKING WITH THE UNION? LABOR UNION MEMBERSHIP, WORKING CONDITIONS, AND POSTRETIREMENT HEALTH IN THE MIDWEST

**DOI:** 10.1093/geroni/igac059.2505

**Published:** 2022-12-20

**Authors:** Michal Engelman, Yue Qin

**Affiliations:** University of Wisconsin-Madison, Madison, Wisconsin, United States; University of Wisconsin-Madison, Madison, Wisconsin, United States

## Abstract

American Employment experiences over the past five decades have been shaped by growing prevalence of bad jobs – those that are precarious and offer few pension or health insurance benefits – and a marked decline in unionization. Previous health research has highlighted the deleterious implications of bad jobs and yielded mixed or inconclusive findings about union membership. However, most of this research focused on working-age adults, and few studies have examined the long-term impacts of working conditions and union membership. We fill this gap via data from the Wisconsin Longitudinal Study – a sample of men and women who graduated from Wisconsin high schools in 1957 and have been followed through their working years, past retirement, and into oldest-old ages. We estimated regression models examining the impact of union participation in 1975 on subsequent self-rated health and depressive symptoms (measured in 1993, 2004, and 2011). Our findings suggest that union participation was associated with poorer self-rated health in 1993 (OR=0.67, 95% CI (0.48, 0.96)), with a stronger negative effect for more active union members (OR=0.58, 95% CI (0.36, 0.96)), even after controlling for socioeconomic status in childhood and adulthood. This effect dissipated by 2004, when most WLS participants were nearing retirement and further diminished by 2011, when participants were in their 70s. We found no significant effects of union activity on depressive symptoms. Job characteristics and the historical decline in the prevalence and power of unions over the cohort’s lifetime provide important contexts for interpreting these results.

